# Genomic-wide identification and expression analysis of R2R3-MYB transcription factors related to flavonol biosynthesis in *Morinda officinalis*

**DOI:** 10.1186/s12870-023-04394-6

**Published:** 2023-08-07

**Authors:** Jingyu Li, Shiqiang Xu, Yu Mei, Yan Gu, Mingyang Sun, Wenting Zhang, Jihua Wang

**Affiliations:** 1 Guangdong Provincial Key Laboratory of Crops Genetics and Improvement, Crop Research Institute, Guangdong Academy of Agriculture Sciences, Guangzhou, 510640 China; 2 Guangdong Provincial Engineering and Technology Research Center for Conservation and Utilization of the Genuine Southern Medicinal Resources, Guangzhou, 510640 China

**Keywords:** *Morinda officinalis*, R2R3-MYB, Transcription factors, Flavonol biosynthesis

## Abstract

**Background:**

The R2R3-MYB transcription factors are a crucial and extensive gene family in plants, which participate in diverse processes, including development, metabolism, defense, differentiation, and stress response. In the Lingnan region of China, *Morinda officinalis* is extensively grown and is renowned for its use as both a medicinal herb and food source. However, there are relatively few reports on the R2R3-MYB transcription factor family in *M.officinalis*.

**Results:**

In this study, we identified 97 R2R3-MYB genes in the genome of *Morinda officinalis* and classified them into 32 subgroups based on phylogenetic comparison with *Arabidopsis thaliana*. The lack of recent whole-genome duplication events in *M.officinalis* may be the reason for the relatively few members of the R2R3-MYB family. We also further analyzed the physical and chemical characteristics, conserved motifs, gene structure, and chromosomal location. Gene duplication events found 21 fragment duplication pairs and five tandem duplication event R2R3-MYB genes in *M.officinalis* may also affect gene family expansion. Based on phylogenetic analysis, *cis*-element analysis, *co*-expression analysis and RT-qPCR, we concluded that *MoMYB33* might modulate flavonol levels by regulating the expression of 4-coumarate-CoA ligase *Mo4CL2*, chalcone isomerase *MoCHI3*, and flavonol synthase *MoFLS4/11/12*. *MoMYB33* and *AtMYB111* showed the highest similarity of 79% and may be involved in flavonol synthase networks by the STRING database. Moreover, we also identified MoMYB genes that respond to methyl Jasmonate (MeJA) and abscisic acid (ABA) stress by RT-qPCR.

**Conclusions:**

This study offers a thorough comprehension of R2R3-MYB in *M.officinalis*, which lays the foundation for the regulation of flavonol synthesis and the response of MoMYB genes to phytohormones in *M.officinalis*.

**Supplementary Information:**

The online version contains supplementary material available at 10.1186/s12870-023-04394-6.

## Background

The MYB transcription factors (TF) are crucial in various biological processes such as plant growth, development, and metabolism, as well as in response to environmental stresses and hormone signaling [1, 2]. The MYB TF contains a highly conserved MYB DNA-binding domain (DBD) in the N-terminal region that is usually composed of 1–4 serial imperfect repeat sequences, each consisting of three α-helices [1, 3]. The second and third helices form a helix-turn-helix (HTH) structure, and the third α-helix is essential for DNA binding, directly interacting with the major groove of the target DNA [4]. Based on their structural characteristics, MYB TFs can be divided into four groups: 1R-MYB, R2R3-MYB, R1R2R3-MYB, and 4R-MYB [5, 6].

The R2R3-MYB genes, among the MYB TF families, have been the most widely studied [1, 7–11]. With the advancement of high-throughput sequencing technologies, R2R3-MYB proteins can be identified on a genome-wide scale in various plant species [1, 12–16]. The number of R2R3-MYBs in plants is typically between 69 and 406, and these genes have mainly evolved through natural selection and genome recombination and amplification [17]. The tetraploid cotton *Gossypium hirsutum* genome has the highest number of R2R3-MYBs (406) [18], while previous studies have identified 285, 244, 157, 134, and 70 R2R3-MYB genes in *Musa acuminata* [19], *Glycine max* [20], *Zea mays* [21], *Vitis vinifera* [22], *Beta vulgaris* [23], respectively. Furthermore, the sweet cherry genome has the lowest number of R2R3-MYB genes, totaling only 69 [24].

In many species, R2R3-MYB transcription factors play a significant role in regulating flavonoid biosynthesis [25]. The S4 subfamily members have been found to have a repressive role in anthocyanin biosynthesis [26]. Members of R2R3-MYB genes of subfamily 5 or 6 serve as a component of the MYB-bHLH-WDR (MBW) transcriptional complex to activate the anthocyanin or proanthocyanidin accumulation [27]. Flavonols in plants are derived from dihydroflavonols through the action of flavonol synthase (FLS). In *Arabidopsis thaliana*, *AtMYB11*, *AtMYB12*, and *AtMYB111* are classified under subfamily 7 (S7) and redundantly regulate the biosynthesis of flavonols through regulation of *AtFLS1* gene expression [28, 29]. Gibberellic acid (GA) inhibits flavonol biosynthesis via DELLA proteins, which interact with SG7 MYBs (*AtMYB12* and *AtMYB111*) and increase the transcriptional levels of the flavonol biosynthesis gene [30]. In *V. vinifera*, the S7 subfamily member *VvMYBF1* acts as a specific activator of *VvFLS1* to promote flavonol accumulation [31]. In addition, miR828 and miR858 regulate *VvMYB114* to promote anthocyanin and flavonol accumulation [32]. In *Fagopyrum tataricum*, *FtMYB6* promotes flavonol biosynthesis by activating flavanone 3-hydroxylase (F3H) and *FtFLS1* expression [33]. *PpMYB15* and *PpMYBF1* are functional flavonol-specific positive regulators in peach fruit [34]. In *Chrysanthemum morifolium*, the heterologous expression of S4 subfamily members *CmMYB1* in *A. thaliana* inhibited the flavonol levels [35]. In *F. tataricum*, the jasmonate-responsive subgroup 4 R2R3-MYB TF *FtMYB13*/14/15/16 (S4) directly repress rutin biosynthesis, *FtMYB13/14/15* repress phenylalanine ammonialyase gene expression, and the importin protein Sensitive to ABA and Drought 2 (*FtSAD2)* and jasmonate ZIM domain 1 (*FtJAZ1)* significantly promote the repressing activity of FtMYBs [36]. In *A.thaliana*, *MYB21* and its homologs *MYB24* and *MYB57*, which belong to subgroup 19, promote flavonol biosynthesis through the regulation of *FLS1* gene expression [28].

*M. officinalis* is a famous medicinal plant widely cultivated in Guangdong Province, China. The roots of *M. officinalis* have been commonly used in tonic products for nourishing the liver and kidneys, dispelling wind and dampness, and enhancing immune function [37]. Flavonoids, which belong to polyphenols, are essential secondary metabolites in plants, including *M. officinalis* [38–42]. Flavanols, flavanones, flavonols, isoflavones, flavones, and anthocyanins are all flavonoid compounds [38, 39]. Flavonol compounds have garnered much attention due to their extensive pharmacological effects. For example, quercetin, a flavonol widely found in fruits, vegetables, and plants, is one of the most powerful natural antioxidants with cardiovascular properties [43]. Morin owns antioxidant, anti-inflammatory, cardioprotective, neuroprotective, anti-diabetic, anti-microbial, and anticancer potentials [44]. Isoquercitrin and isohyricoside may protect the myocardium by protecting cell membranes from oxidative damage [45].

As described above, R2R3-MYB genes are crucial in regulating flavonol synthesis in other plant species. Flavonols are also essential secondary metabolites in the roots of *M. officinalis*. However, the study of R2R3-MYB genes of *M. officinalis* was rare. The *M. officinalis* high-quality genome was assembled and annotated by our research group [40], which could be the basis for more detailed analyses. In addition, *Ophiorrhiza pumila*, a medicinal plant belonging to the Rubiaceae family like *M.officinalis*, has recently completed genome assembly at the chromosome level [46]. In this study, the model plants *A. thaliana* and *O. pumila* were selected for collinearity analysis. We obtained more comprehensive information on the R2R3-MYB gene family from the genome of *M. officinalis*. A total of 97 MoMYB genes were identified using bioinformatics. And the phylogenetic relationship, conserved motifs, gene structure, chromosomal location, gene duplication, collinearity, *cis*-element, and expression trends of MoMYB genes were analyzed. To predict the network of MYB-regulated flavonol synthesis, the *Co*-expression analysis between MoMYB genes of flavonol synthesis-related subfamilies, essential structural genes in flavonol synthesis, and the content of flavonol metabolites was performed. Furthermore, we also identified some MoMYB genes in response to MeJA and ABA. This study provided the fundamental basis for the further functional investigation of MoMYB genes.

## Results

### Identification and analysis of R2R3-MYB transcription factor in *M. officinalis*

Using BLASTP and analysis searches, 242 MYB candidate genes were identified from the *M. officinalis* genome (NCBI accession number: ASM2008022v1) [40]. Using the Pfam database, the number of MYB DNA-binding domain repetitions of MYB candidates was confirmed. The MYB candidates with two MYB DNA-binding domain repeats were verified by SwissProt and Scanprosite and determined to be R2R3-MYB genes. Finally, 97 R2R3-MYB genes were identified in the *M. officinalis* genome (Table S1). To simplify the name, all found R2R3-MYB genes were given the prefix "Mo" for *M.officinalis* and were numbered according to their chromosomal location, resulting in *MoMYB1* through *MoMYB97*. Analyzing the physical and chemical properties of MoMYB found that the lengths ranged from 106 to 1063 aa, the molecular weights were between 12.22 kDa to 119.11 kDa, and the predicted pI values ranged from 4.85 to 10.62 (Table S1). Additionally, the majority of MoMYB proteins were predicted to be localized to the nucleus (Table S1).

**Fig. 1:**
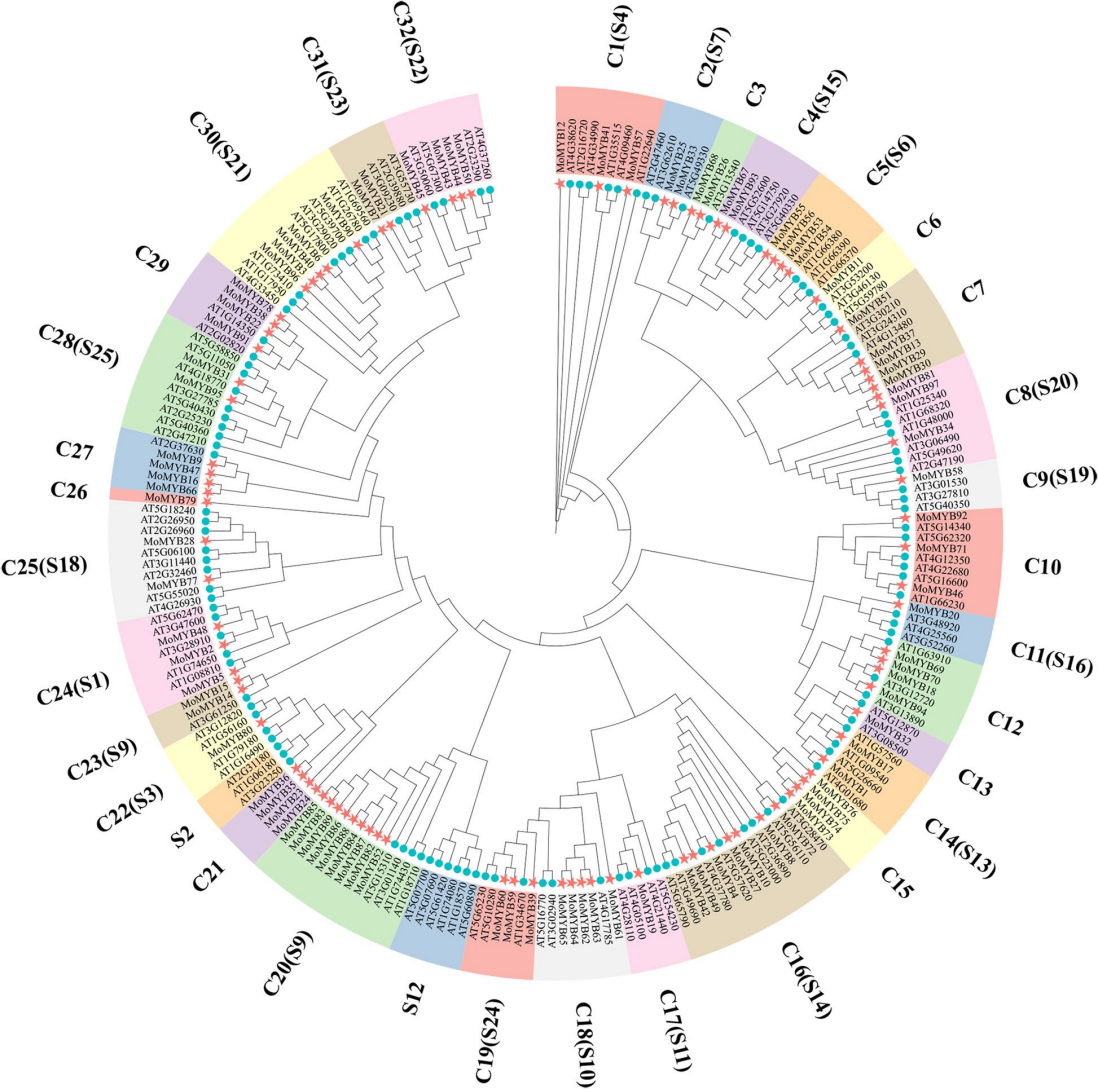
Phylogenetic trees of MoMYB and AtMYB proteins. The phylogenetic tree was created using IQ-tree with 1000 bootstrap replications. The model was Q.insect + R5. The Phylogenetic tree is divided into 32 subfamilies, labelled with protein gene names in different background colours, with the corresponding subfamily names marked in the outer circle. The inner circles are indicated by blue and red circles for Arabidopsis and *M. officinalis* MYB proteins respectively

To study the evolutionary relationship among the MoMYB and AtMYB genes, we constructed the phylogenetic tree by using the conserved MYB domain sequences. Referring to the grouping of *A. thaliana*, the MoMYB proteins were divided into 32 subgroups (C1 to C32), as shown in Fig. 1. Remarkably, 20 of the total 32 subgroups contained known groups of AtMYB genes from previous studies, e.g., C1 (S4), C2 (S7), C4 (S15), C5 (S6), etc.

The conserved motifs and exon–intron structure of MoMYB were exhibited in Fig. S1. As exhibited in Fig. S1, all MoMYB genes except *MoMYB43* had highly conservative motif 2, motif 1, and motif 3. the arrangement of motif 2, motif 1, and motif 3 is also conserved in the C-terminal of MYB proteins. According to the results of the exon–intron distribution, the number of exons varies from 1 to 12. As expected from previous studies, Most MoMYB genes have a structure of three exons and two introns (68 out of 97 MoMYB genes, 70%) (Fig. S1).

### Chromosomal location of MoMYB genes

**Fig. 2:**
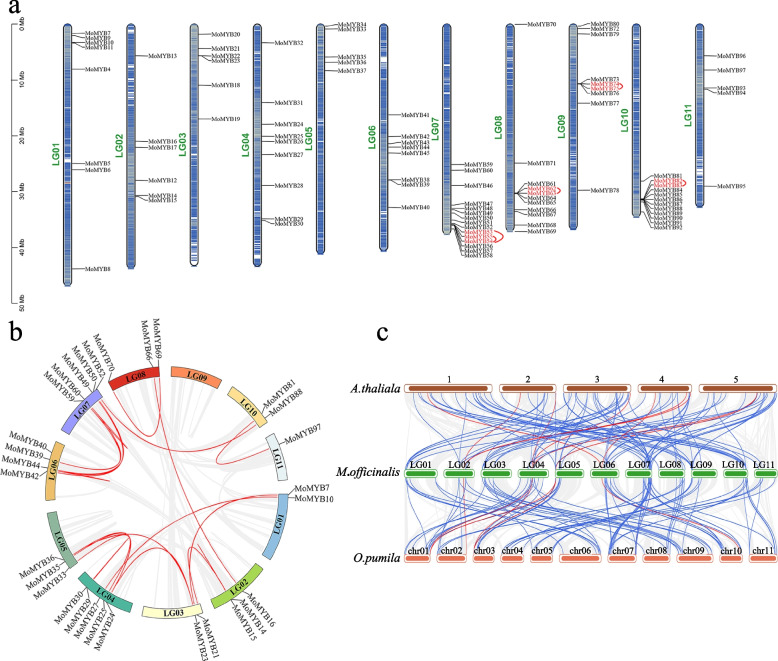
Chromosomal location and Synteny analyses of MoMYB genes. **a** Chromosomal location. The red line group indicates tandem duplication. **b** Schematic representations for the interchromosomal relationships of *M. officinalis* R2R3-MYB genes. Gray lines and red lines represent all the synteny gene pairs and MoMYB gene pairs, respectively. **c** Synteny analyses of the R2R3-MYB genes between *M. officinalis* and two representative species (*A. thaliana* and *O. pumila*). Gray lines represent the collinear blocks in the genomes of *M. officinalis* and other plant species, blue lines emphasize the syntenic MoMYB genes pairs, and red lines emphasize the syntenic five candidate MYB genes pairs

Analyzing the chromosomal location of each MoMYB gene found that 94 MoMYB genes were dispersed on 11 chromosomes of *M. officinalis* (Fig. 2a), and the other 3 genes were located on 3 scaffolds. MoMYB genes were unevenly located, with the largest number of MoMYB genes on the chromosomes LG07 (15 genes) and the fewest number on chromosomes LG05 and LG11 (5 genes each). The distribution of gene density is demonstrated within the chromosome.

### The evolution of R2R3-MYB gene family in *M. officinalis*

Gene duplication events mainly drove the evolution of multigene families. In this study, BLASTP and MCScanX were used to identify the duplicate genes of the MoMYB gene family in the *M. officinalis* genome. Four pairs of tandem duplicates were detected on chromosomes LG07, LG08, LG09, and LG10: *MoMYB53*/*54*/*55*, *MoMYB62*/*63*, *MoMYB74*/*75*, and *MoMYB82*/*83* (Fig. 2a). Intrachromosomal duplications of MoMYB genes were shown by the red line in Fig. 2b. In detail, 21 gene pairs of segmental duplications were identified on all 11 chromosomes (Table S2). It is worth noting that *MoMYB33* and *MoMYB25*, members of the S7 subfamily, are a pair of segmental duplication genes.

To analyze the evolutionary gene family size of the R2R3-MYB of *M. officinalis*, we compared and analyzed the R2R3-MYB gene of *M. officinalis* and other Rubiaceae plants. We identified R2R3-MYB proteins in *O. pumila* and *Coffea canephora*, which have 78 and 79 R2R3-MYB genes, respectively. We constructed the phylogenetic tree of R2R3-MYB proteins from *M. officinalis*, *O. pumila*, *C. canephora* and *A. thaliana*. The Phylogenetic tree is divided into 34 subfamilies (Fig. S2, Table S3). In most subfamilies, these three species without recent genome-wide replication events have a low number of members, and MoMYB proteins may have gene loss only in subfamilies 11 and 15 (Fig. S2, Table S3). Gene duplication events may affect the size of the MYB gene family. For example, the number of members of the subfamily 16 is higher than that of the other two closely related species, which may be caused by tandem duplicates of *MoMYB53*/*54*/*55*.

To further understand the evolutionary relationship of R2R3-MYB members in different plant species, we constructed the comparative synteny maps of *M. officinalis* association with two representative species, including *A. thaliana* and *O. pumila* (Fig. 2c). In total, MoMYB genes displayed a syntenic relationship with those in *A. thaliana* (89) and *O. pumila* (96) with blue lines (Table S4, Fig. 2c). The number of R2R3-MYB genes in *O. pumila* is far less than that in *A. thaliana*, but there are more syntenic gene pairs between *M. officinalis* and *O. pumila*, indicating that there is a potential evolutionary process between them.

In Fig. 2c, we have marked with red lines the syntenic pairs of members of the S4 and S7 subfamilies of *M. officinalis* and two other species, e.g., *MoMYB12*, *AtMYB6/7/32* and *Opuchr02_g0076850-1.1*; *MoMYB25*, *AtMYB11/12/111*, *Opuchr01_g0075220-1.1* and *Opuchr03_g0001460-1.1*; *MoMYB33*, *AtMYB111*, *Opuchr01_g0075220-1.1,* and *Opuchr03_g0001460-1.1*; *MoMYB41*, *AtMYB3/4,* and *Opuchr10_g0057590-1.1*; *MoMYB57* and *Opuchr07_g0084770-1.1.* It can be found that the syntenic gene pairs of *M. officinalis* and *A. thaliana* belong to the same subfamily and may have functional conservation.

### Expression patterns of MoMYB genes

**Fig. 3:**
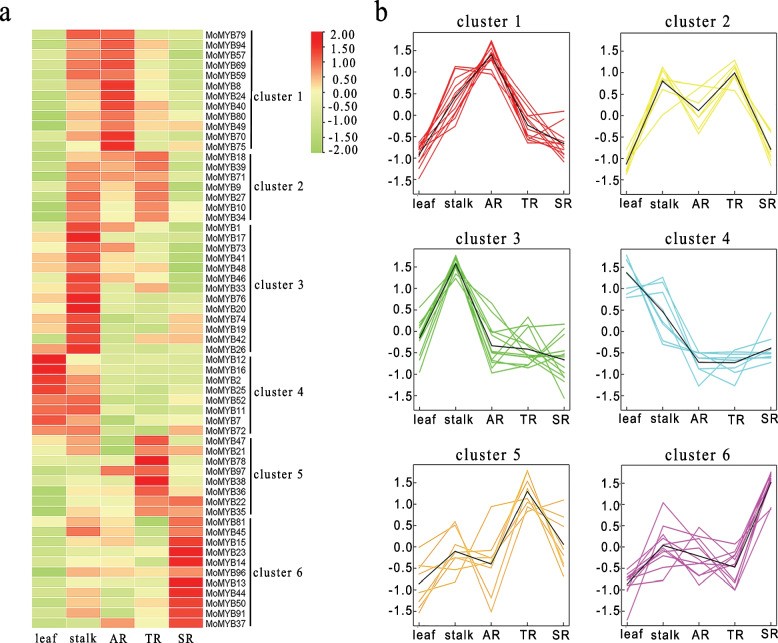
The expression patterns of *M. officinalis* R2R3-MYB genes. **a** The heatmap of expression patterns of MoMYB genes. The expression levels are illustrated in green–red scale. Green indicates lower expression and red indicates higher expression. **b** Clustering expression of MoMYB genes based on their tissue-specific expression. leaf, one-year-old leaf; stalk, one-year-old stalk; AR, one-year-old root; TR, three-year-old root; SR, six-year-old root

To characterize the expression of MoMYB genes, we analyzed RNA-Seq data of 97 MoMYB genes in five different tissues, including stems, leaves, one-year-old roots (AR), three-year-old roots (TR), and six-year-old roots (SR). Out of the 97 MoMYBgenes, 70% were expressed in at least one tissue, with a broad expression range with a maximum of 1–564 FPKM (FPKMmax) (Fig. 3a and Table S5). The remaining 30% of MoMYB genes showed a low expression with an FPKMmax < 1 and were inferred not expressed. The highest number of MoMYB genes abundantly expressed in the stalk can be found in Fig. 3a. For further analysis, the expressed MoMYB genes were clustered into 6 groups according to expression modules (Fig. 3b). The cluster 1 genes were abundantly expressed in the stalk and AR, and the expression in roots gradually decreased with increasing growth time; the cluster 2 genes were highly expressed in the stalk, AR and TR, and their expression in roots increased and then decreased with increasing growth time; All genes in cluster 3 were most abundantly expressed in stems; the cluster 4 genes were more expressed in leaves and stalk; the genes in clusters 5 and 6 were more abundantly expressed in TR and SR, respectively. The expression trends of genes in cluster 5 in the three roots were similar to those in cluster 2. The expression abundance of MoMYB genes was tissue-specific in *M. officinalis*. The expression patterns of MoMYB genes in different tissues will provide a reference for the functional study of MoMYB.

### Identification of *Cis-*elements in MoMYB and flavonoid biosynthesis genes

The *Cis-*acting elements contained in the promoter region of the gene are important references for predicting that the gene may participate in specific biological regulatory networks. Therefore, we extracted 2000 bp upstream sequences of the R2R3-MYB genes and flavonoid biosynthesis genes to predict *Cis-*acting elements by PlantCARE. To analyze the possible regulatory mechanism of R2R3-MYB in hormone response, we selected MeJA, ABA, GA, SA, and auxin-related *Cis-*acting elements from among the numerous *Cis-*acting elements for visualization and analysis (Fig. S3a). Among the hormone *Cis-*acting elements contained in R2R3-MYB genes, MeJA-responsive elements (CGTCA-motif and TGACG-motif) and ABA-responsive elements (ABRE) were the most numerous, with 237 and 220, respectively. In addition, the numbers of GA (GARE-motif, TATC-box, and P-box), SA (TCA-element), and auxin (AuxRR, AuxRE, TGA-element, TGA-box) response elements were 82, 63, and 56, respectively. The presence of multiple identical hormone response elements in the promoter region of a gene often means that the gene can respond rapidly and powerfully to the regulation of the hormone. e.g., *MoMYB33* contains four ABA elements, and *MoMYB57* contains four MeJA elements. These results suggest that R2R3-MYB TFs are highly likely to respond to hormonal stress.

R2R3-MYB transcription factor has an essential role in the regulation of flavonoid biosynthesis. The promoter sequences of genes encoding key enzymes in the flavonoid biosynthesis pathway of *M. officinalis* were analyzed to identify MYB binding sites (Fig. S3b). MBS, MRE, MBSI, CCAAT-box, CCGTTG, CAACAG, TAACCA, TAACTG, and CAACCA *Cis-*elements were identified. The gene promoter region contains many MYB binding sites, indicating that MYB genes are more likely to recognize and regulate its expression level, e.g., *MoFLS1* (11), *MoPAL3* (11), *MoCHI1* (10), *Mo4CL2* (10), *MoCHI5* (9), *MoFLS3* (9), *MoPAL1* (9), *MoPAL2* (9) and *MoPAL6* (9). These results suggest that R2R3-MYB TFs may directly target these pathway genes affecting flavonoid biosynthesis.

### Identification of R2R3-MYB was related to flavonol biosynthesis in *M. officinalis*

**Fig. 4:**
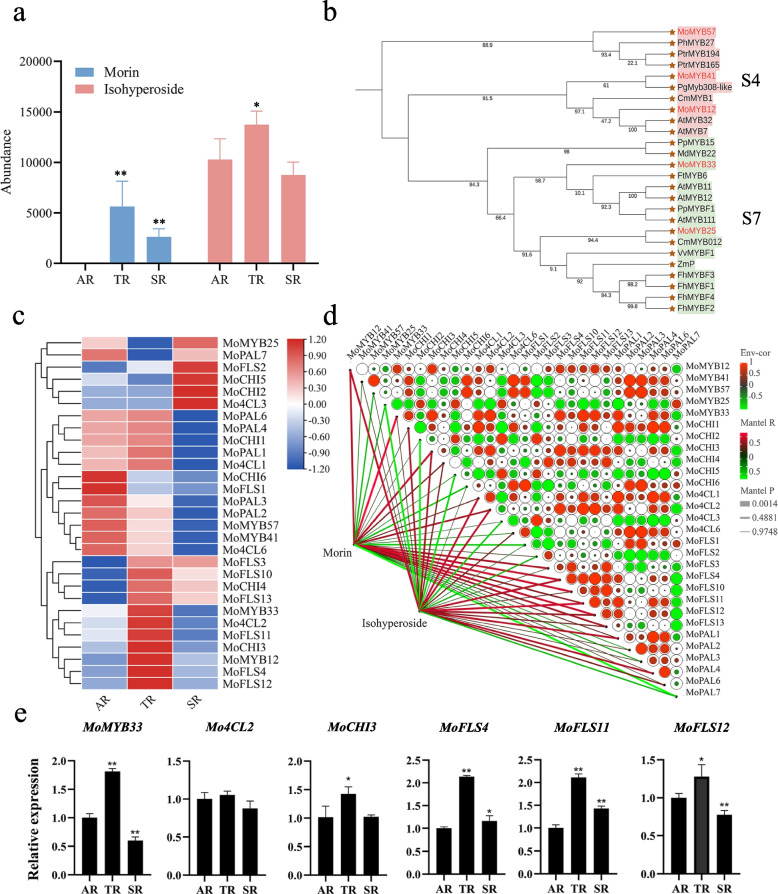
Prediction of flavonol-related MYB gene analysis. **a** The contents of flavonol morin and isohyperoside in roots. **b** Evolutionary tree of flavonol-related MYB genes. **c** Heatmap of the expression patterns of the five *MoMYB* genes and flavonol biosynthesis genes. The expression levels are illustrated in blue-red scale. Blue indicates lower expression and red indicates higher expression. **d** Co-expression analysis of five *MoMYB* genes, flavonol biosynthesis genes, and flavonol metabolites in AR, TR, and SR samples. Red represents a positive correlation, and the green represents a negative correlation. The upper right side is the candidate MYB gene and flavonol synthesis-related genes. The color of each color block in the figure indicates the positive or negative correlation coefficient between genes, and the size of the color block indicates the absolute value of the correlation coefficient. The lower left side is the association data between flavonol content and genes. The color of the line indicates the strength of the correlation, the thickness of the line indicates the degree of significance, and the thicker the line, the higher the significance. Phenylalanine ammonialyase (PAL), chalcone isomerase (CHI), 4-coumarate-CoA ligase (4CL), chalcone synthase (CHS), flavonol synthase (FLS). **e** RT-qPCR. Statistically significant differences were determined by t-test (* *p* < 0.05, and ** *p* < 0.01)

Flavonol synthase is the essential enzyme in the first step of the flavonoid precursor into the flavonol branching pathway. In this study, transcriptome sequencing analysis revealed that seven of the 8 expressed FLS genes were up-regulated in TR compared to AR (Table S6). Notably, the flavonol morin and isohyperoside were also up-regulated, and all differentially expressed flavones and isoflavones were significantly down-regulated in TR compared to AR by metabolomics analysis (Fig. 4a and Table S7). Studies in other plant species have shown that R2R3-MYB genes of the S7 and S4 subfamilies regulated the expression of FLS and some other key enzymes in flavonol biosynthesis [9, 47]. Therefore, we hypothesize that MoMYB may affect flavonol accumulation by regulating some key enzymes. In *M. officinalis*, *MoMYB12*, *MoMYB41*, and *MoMYB57* in the S4 subfamily and *MoMYB25* and *MoMYB33* in the S7 subfamily were selected to be candidates for genes regulating the flavonol biosynthesis. Then, the phylogenetic tree was generated, including 5 candidate MoMYB genes and 13 R2R3-MYB genes regulating flavonol biosynthesis in other species (Fig. 4b). In the S4 subgroup, *MoMYB57*, *PhMYB27*, *PtrMYB194*, and *PtrMYB165* clustered together. *MoMYB12* and *MoMYB41* clustered with *PgMYB3008-like*, *CmMYB1*, *AtMYB32*, and *AtMYB7*. In S7 subgroup, *MoMYB33* clustered with *FtMYB6*, *AtMYB11*, *AtMYB12*, *PpMYBF1*, and *AtMYB111*. Furthermore, *MoMYB25* was highly close to *CmMYB012*.

To analyze the gene expression of candidate genes and the flavonol biosynthetic pathway genes, we identified the flavonol biosynthetic pathway genes of *M. officinalis*, including *MoPAL*, *Mo4CL*, *MoC4H*, *MoCHS*, *MoCHI*, *MoFLS,* and *MoF3'H* (Table S6). Then, a heatmap of transcriptome data of five expressed candidate MoMYB genes and some important pathway genes was performed in Fig. 4c.

**Fig. 5:**
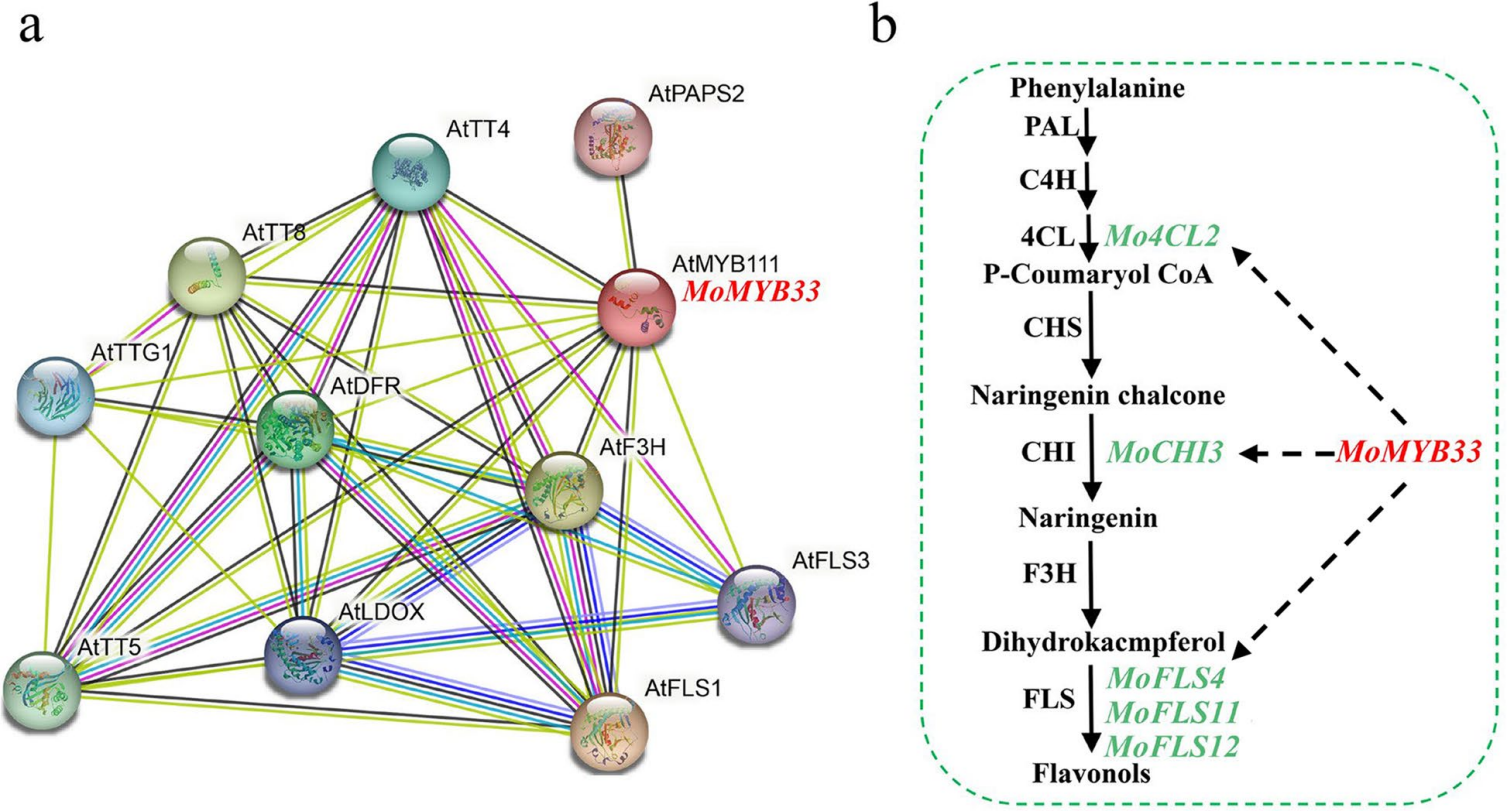
The possible model for the regulation of flavonol accumulation by *MoMYB33.***a** Interaction network analysis of *MoMYB33* by STRING 11.5. The reference species is Arabidopsis. **b** Predicted regulation of *MoMYB33* in the flavonol pathway

Predicting the possible network of MoMYB-regulated flavonol metabolism, a correlation analysis between 5 candidate MoMYB *genes*, 24 important flavonol biosynthetic enzymes, and flavonoid metabolite content was performed (Fig. 4d and Table S8). The result revealed that *MoMYB12, MoMYB41, MoMYB57, MoMYB25*, and *MoMYB33* were correlated with flavonol synthesis genes with absolute values greater than 0.7 in 15, 14, 11, 16, and 7 pairs, respectively. Of these, four and one genes had correlation coefficients greater than 0.7 with metabolite morin and isohyperoside. expression, respectively (Fig. 4d and Table S8). Based on previous literature reports that S4 and S7 subfamily members are negative and positive regulators of flavonols, respectively, and the trend of flavonol level accumulation in *M.officinalis* roots, we inferred that *MoMYB33* may be a flavonol regulator. Moveover, we also found that *MoMYB33* and *Mo4CL2*, *MoCHI3* and *MoFLS4/11/12* clustered in the same branch in the expression heat map, and *MoMYB33* also had high expression correlation with these genes, with correlation coefficients greater than 0.9 (Fig. 4c/d and Table S8). MoMYB33 and morin and isohyperoside levels were also correlated. Analysis of the results of RT-qPCR revealed that *MoMYB33* and *Mo4CL2, MoCHI3 and MoFLS4/11/12* also showed similar expression trends (Fig. 4e). The *MoMYB33* protein interactions in *M.officinalis* studied by STRING online database (Fig. 5a). *A.thaliana* was selected as the reference species. *MoMYB33* and *AtMYB111* showed the highest similarity of 79% and may be involved in powerful cross-linking networks. This network largely participated in flavonol synthase pathways because of most of the important factors like *AtMYB111*, *AtFLS1*, *AtFLS3*, *AtF3H*, *AtDFR*, *AtTT4*, *AtTT5*, *AtLDOX*, and *AtTTG1*. According to functional predictions and *Co*-expression analysis, *MoMYB33* might affect flavonol accumulation in *M. officinalis* through the regulation of *Mo4CL2*, *MoCHI3*, *MoFLS4/11/12* (Fig. 5b).

### Expression patterns of MoMYB genes under hormonal treatments

Plant hormones induce the accumulation of a variety of secondary metabolites via the regulation of hormone-responsive transcription factors. A total of 8 MoMYB genes, which were phylogenetically close to the known phenylpropanoid metabolism-related *MYB* proteins in *A. thaliana*, have been selected for research of expression patterns in response to MeJA and ABA treatments in one-year-old roots using RT-qPCR. As shown in Fig. S4, the expression of all genes changed significantly after the three hormone treatments. MeJA treatment significantly induced the expression levels of all 8 MoMYB *genes* at Me1h, with the expression of *MoMYB29*, *MoMYB30*, *MoMYB33*, and *MoMYB57* down-regulated and *MoMYB41* up-regulated at Me6h. Besides, the expression levels of *MoMYB41* were highest at Me12h, and *MoMYB97* was high at all four treatment time points. Under ABA treatment, the expression of *MoMYB34*, *MoMYB41*, *MoMYB57*, *MoMYB81*, and *MoMYB97* was significantly up-regulated after 1 h. In addition, the expression of *MoMYB33* was first down-regulated at A1h and A6h and then up-regulated at A12h. These above findings might be valuable for studying the regulatory function of MoMYB genes under hormonal treatments.

## Discussion

*M. officinalis*, one of the "Four Southern Herbs" in Guangdong, China, contains several active substances with medicinal properties [48, 49]. Flavonol is one of the important active substances in *M. officinalis* roots, and the R2R3-MYB gene was found to regulate flavonol accumulation in many other plants [40]. However, there are few studies on the MoMYB genes. This study is the first to identify and annotate the MoMYB gene family based on the genome. Here, a total of 97 MoMYB genes were identified in *M. officinalis*. Compared to *G. hirsutum* (406), *M. acuminata* (285), *A. thaliana* (126), *Ananas comosus* (184), *Z. mays* (188), and *Solanum tuberosum* (111), the number of the R2R3-MYB gene family was significantly less in *M. officinalis* [1, 18, 19, 50–52]. *M.officinalis* genome assembly has high completeness and consistency [40], and identifying R2R3-MYB gene family members is relatively complete and reliable. High numbers of R2R3-MYB genes are primarily attributed to ancient whole genome duplication events, as observed in several plants, including *A. thaliana*, *O. sativa*, *Populus trichocarpa*, *G. max*, and *Malus x domestica* Borkh [53–56]. Genome evolution analysis found that *M.officinalis* has no whole-genome duplication event recently [40], which may be the reason for the relatively few members of the R2R3-MYB gene family. Interestingly, the close relatives of *M. officinalis*, *O. pumila* and *C. canephora*, which also belong to Rubiaceae, have also no whole-genome duplication event [46], and only contain 78 and 79 members of the R2R3-MYB gene family. The comparative analysis of the phylogenetic tree of the R2R3-MYB proteins of the three Rubiaceae species found that most of the subfamilies had fewer members than *A. thaliana*, but only two subfamilies in *M. officinalis* experienced complete gene loss evolution. Whole genome duplication events may be an important factor affecting the size of R2R3-MYB family members in Rubiaceae. In addition, Stracke et al. also speculated that only 70 R2R3-MYB genes in sugar beet were related to the absence of whole genome duplication [23]. Gene duplication events also affect gene family size [57]. For example, the generation of *MoMYB53/54/55* tandem repeat pair may be the reason why the number of genes in this subfamily of *M. officinalis* is much higher than that of the other two Rubiaceae species.

Collinearity analysis revealed striking similarities between syntenic orthologous groups and phylogenetic relationships, and also verified the closer close relationship between related species, providing a reference for the study of gene function in new species.

Previous research demonstrated that the R2R3-MYB gene family exhibits significant conservation and diversification in the plant kingdom [21]. Phylogenetic analysis and reference to the subfamily classification of *A. thaliana* revealed that the MoMYB genes could be divided into 32 subfamilies (C1-C32). Moreover, the physical and chemical properties of MoMYB protein members were varied, such as the lengths, molecular weight, and pI value, suggesting a high degree of diversity among these *MoMYB* members. Some studies have found that the R2R3-MYB genes clustered in the same subfamily in different species have specific functional conservation. For example, members of the S7 subfamily positively regulate flavonol synthesis in different species, including *AtMYB11/12/111* in Arabidopsis, *VvMYBF1* in *V. vinifera*, *PpMYB15* and *PpMYBF1* in Peach, *FeMYBF1* in Buckwheat, and *Malus sieversii MYB22* gene [29, 31, 34, 36, 58–60]. In the S4 subfamily, the *VvMYB114* of *V. vinifera* negatively regulated flavonol synthesis [61]. In *F. tataricum*, *FtMYB13*/14/15/16 (S4) directly represses rutin biosynthesis [36]. Moreover, collinearity analysis also found that members of the *M.officinalis* S7 and S4 subfamily have a collinear relationship with members of the S7 and S4 subfamily in Arabidopsis. Therefore, two S7 subfamily members (*MoMYB25* and *MoMYB33*) and three S4 subfamily members (*MoMYB12*, *MoMYB41*, and *MoMYB57*) were selected as candidate genes possibly regulating flavonol synthesis.

In *Prunus persica*, sequence analysis revealed that *PpMYB15* and *PpMYBF1* belonged to the flavonol regulatory subfamily and were highly correlated with flavonol content and *PpFLS1* gene expression, and further experiments also revealed that they could activate the promoter region of *PpCHS1*, *PpCHI1*, *PpF3H* and *PpFLS1* and positively regulate flavonol accumulation [34]. Naik et al. also identified the flavonol regulator *MtMYB134* by evolutionary tree analysis and found a high correlation with both *MtFLS2* gene expression and flavonol metabolite levels, and overexpression of *MtMYB134* in hairy roots of *Medicago truncatula* promoted the biosynthesis of various flavonol derivatives [62]. In this study, We also identified *MoMYB33*, a member of the flavonol regulator subfamily S7. It was homologous to the validated flavonol regulators of other species, and *MoMYB33*, *Mo4CL2, MoCHI3, MoFLS4/11/12* and the flavonol morin content, were correlated. RT-qPCR also revealed that the expression trends of *MoMYB33* and *Mo4CL2, MoCHI3, MoFLS4/11/12* were consistent. By STRING database analysis, *MoMYB33* was most similar to the vital flavonol regulator *AtMYB111* in Arabidopsis with 79% similarity. Multiple MYB-binding cis-elements were also present in the promoter region of the *Mo4CL2, MoCHI3, MoFLS4/11/12* gene. Therefore, we speculate that *MoMYB33* may affect flavonol accumulation by regulating *Mo4CL2, MoCHI3, MoFLS4/11/12* genes in *M. officinalis*.

Hormones are essential stressors stimulating the synthesis of secondary metabolites, including flavonol metabolites [36]. In a previous study, the expression of the flavonoid synthetase was activated by MeJA treatment, and several novel MYB candidates may regulate flavonol synthesis in Pear [63]. In this study, MeJA treatment significantly induced the expression levels of 8 MoMYB genes after 1 h of MeJA treatment. Studies on the relationship between ABA and flavonols found that ABA metabolism could activate flavonol metabolisms, and flavonol quercetin also regulated the ABA signaling pathway [64, 65]. Overexpression of *SbMYB2* or *SbMYB7* in *Scutellaria baicalensis* promoted phenylpropanoid accumulation and enhanced ABA stresses tolerance in transgenic tobacco [66]. In this study, the expression level of 8 MYB genes was affected by ABA treatment. These results will provide a reference for studying the MYB response hormone regulatory network.

## Conclusion

In summary, we identified 97 MoMYB proteins in *M. officinalis* and conducted comprehensive analyses on their physical properties, evolutionary relationships, conserved motifs, exon–intron structures, chromosomal location, gene duplication, syntenic relationship, expression patterns, cis-acting elements, *Co*-expression, RT-qPCR, and hormone treatments. Based on these analyses, we found that the S7 subfamily gene *MoMYB33* likely regulates flavonol accumulation by regulating *Mo4CL2, MoCHI3, MoFLS4/11/12*. In addition, we also identified some MoMYB genes that responded to MeJA and ABA hormone stress. These findings provide a theoretical foundation for future studies aimed at exploring the functional characteristics of R2R3-MYB genes in *M. officinalis*.

## Methods

### Identification members of R2R3-MYB gene family

The *M. officinalis* genome can be downloaded from the *M. officinalis* database (http://morindaofficinalisgenome.com/download.php) and NCBI (Accession number: ASM2008022v1) [40]. The *O. pumila* genome downloaded from the DDBJ database (accession no. BLIW01000001-BLIW01000013). The *C. canephora* genome (AUK_PRJEB4211_v1) downloaded from the Ensembl database. In order to find R2R3-MYB genes from the *M. officinalis, O. pumila and C. canephora* genome, the *A. thaliana* R2R3-MYB proteins were acquired from the Ensemble Plants database and used as the query for a BLASTP search. Meantime, the MYB DNA-binding domain (PF00249) was obtained from the Pfam database, and it was then utilized to use the Hmmsearch tool in HMMER 3.0 to identify the MYB genes from the *M. officinalis* genome. The cutoff value was set to 1e^−5^, and the default values were used. Furthermore, the MYB protein sequences identified by both above methods were integrated. We examined the putative MYB sequences for the presence of the R2R3-MYB domain using the Pfam and PROSITE databases.

### Phylogenetic analysis and classification of the MoMYB proteins

The protein sequences of MoMYB and AtMYB proteins were compared by Muscle. After comparison, the sequence was used to construct a phylogenetic tree using the IQ-tree maximum likelihood method with the parameters Q.insect + R5 model and 1000 bootstrap replications. Then, the members of the phylogenetic tree were classified into subgroups based on the AtMYB protein classification. The phylogenetic tree of the 97 MoMYB proteins was adopted with the same method. Interactive Tree of Life (iTOL) was used to view and embellish the phylogenetic tree.

### Analysis of the characteristics of MoMYB genes

The essential properties of MoMYB proteins were predicted by ExPASy, including molecular weights (MW) and the theoretical isoelectric point (PI). In addition, the subcellular localization of MoMYB genes was predicted by the CELLO tool. The genomic sequences and GFF3 file of *M. officinalis* were utilized by TBtools to display the exon–intron structure of these genes. With a maximum of 10 motifs, the program MEME predicted the conserved motifs of MoMYB proteins. The motifs with an e-value lower than 1e^−10^ were retained for further analysis. Tbtools was used to show the phylogenetic tree, conserved motifs, and gene structures of MoMYB protein sequences [67].

### Chromosomal distribution and synteny analysis of MoMYB genes

The reference *M. officinalis* genome information was used to determine the chromosomal positions of the MoMYB genes [40]. MCScanX was used to evaluate the gene duplication events [68]. The tandem duplicate gene pairs were located within 200 kb and adjacent to the same chromosomal. The syntenic relationship was determined by the Dual Systeny Plotter software of Tbtools between the R2R3-MYB genes of *M. officinalis*, *A. thaliana*, and *O. pumila*, respectively. These results were visualized using TBtools.

### Plant materials and hormone treatment

RNA-seq and metabolomics was performed using *M. officinalis* leaf, stem, one-year-old roots (AR), three-year-old roots (TR), and six-year-old roots (SR) samples collected from previous studies in our laboratory and stored at -80 °C in the refrigerator [40]. The *M. officinalis* "Gaoji 3" seedlings used for RT-qPCR in this experiment were grown in the herbal greenhouse of Guangdong Academy of Agricultural Sciences for processing and collection. For MeJA and ABA treatment, one-year-old *M.officinalis* "Gaoji 3" seedlings were cultivated in Hoagland's nutrient solutions with 10 μM MEJA and 10 μM ABA, respectively. The roots of *M. officinalis* seedlings were collected at 0 h, 1 h, 6 h, and 12 h after MeJA and ABA treatment, and the roots were also collected at 24 h after MeJA treatment. After collection, all plant samples were frozen in liquid nitrogen and preserved at -80 °C.

### *Cis-*elements of promoters analysis

The *Cis*-elements of promoters of MoMYB genes and flavonoid biosynthesis genes were predicted by PlantCARE. This result processing and image beautification was performed through TBtools.

### RNA-Seq expression analysis

The *M. officinalis* tissue samples (stem, leaf, AR, TR, and SR) were sent to Wuhan MetWare Biotechnology Co., Ltd. for transcriptome sequencing. There are three biological repeats in each tissue sample. RNA-Seq data were obtained from previous studies in our lab, and the specific sequencing methods and data analysis have been published in previous articles [40]. The transcriptional expression profile of MoMYB genes was displayed by TBtools. The expression trends of MoMYB genes were clustered by R software.

### Broad untargeted metabolic profiling

The metabolites of *M. officinalis* Root (AR, TR, and SR) were determined by broad untargeted metabolomics. There are three biological repeats in each sample. The freeze-dried root was crushed using a mixer mill (MM 400, Retsch) with a zirconia bead at a frequency of 30 Hz for 1.5 min. A total of 100 mg of powder was weighed and subjected to overnight extraction at 4 °C using 0.6 ml of 70% aqueous methanol. After centrifugation at 10,000 g for 10 min, the resulting extracts were absorbed onto a CNWBOND Carbon-GCB SPE Cartridge (250 mg, 3 ml; ANPEL, Shanghai, China) and subsequently filtered through an SCAA-104 filter with a pore size of 0.22 μm (ANPEL, Shanghai, China) prior to UPLC-MS/MS analysis.

The UPLC-ESI–MS/MS system (UPLC, Shim-pack UFLC SHIMADZU CBM30A system; MS, Applied Biosystems 4500 Q TRAP) was employed for analyzing the sample extracts. The Analyst v1.6.3 software was used, and the ion spray voltage was set at 5500 V for the positive ion mode and -4500 V for the negative ion mode. The remaining HPLC conditions, including the linear ion trap and triple quadrupole (QQQ) scans and experiments, were performed as previously described, ensuring consistency with our previous study [69].

### *Co*-expression analysis of MoMYB genes, flavonol synthesis genes, and flavonol metabolites

The correlation analysis among candidate MoMYB genes, flavonol synthesis genes, and flavonol metabolite contents was performed via Intergroup correlation tools in Omicshare (https://www.omicshare.com/tools/Home/Soft/ica). The pearson correlation analysis was used. Correlation significance p < 0.05, representing a significant correlation, and p < 0.01, representing a highly significant correlation. A correlation coefficient R greater than 0.6 indicates a strong correlation, 0.4 to 0.6 indicates a moderate correlation, and less than 0.4 indicates a weak or no correlation. The correlation was displayed by Dynamic Network Heat Map tools in Omicshare (https://www.omicshare.com/tools/home/report/report_heatnetwork.html).

### RNA isolation and RT-qPCR

Following the manufacturer's instructions, total RNA was extracted from the roots of *M. officinalis* using the RNAprep Pure Plant Plus Kit DP441 (TIANGEN, China). The RNA was monitored by gel electrophoresis. RT-qPCR was conducted on CFX96 (Bio-Rad, CA, USA) using TB Green Fast qPCR Mix RR430A (Takara Bio, Inc, Japan). The *MoGAPDH* gene was utilized as the reference [70]. The comparative Ct (2^−△△Ct^) method was used to calculate the relative abundance. The RT-qPCR primers are listed in Table S9.

### Supplementary Information

**Additional file 1:** **Figure S1.** The phylogenetic tree, conserved motifs, and exon-intron structure of MoMYB proteins. **Figure S2.** Phylogenetic tree of R2R3-MYB proteins from *M. officinalis*, *O. pumila*, *C. canephora* and *A. thaliana*. **Figure S3.** The Cis-element analysis. **Figure S4.** RT-qPCR of the expression profile of MoMYB genes under hormonal treatments.

**Additional file 2:** **Table S1.** Detailed characteristics of the 97 *M.officinalis *R2R3-MYB proteins in this study. **Table S2.** Segmentally and tandemly duplicated R2R3-MYB gene pairs in *M.officinalis*. **Table S3.** Distribution of subfamily members of the phylogenetic tree of the R2R3-MYB protein from* M. officinalis, O. pumila, C. canephora, *and *A. thaliana. ***TableS4.** The syntenic MoR2R3-MYB gene pairs in *A.thaliana*, *C.canephora*, and *O.pumila*. **Table S5.** RNA-seq data of MoR2R3-MYB in this study. **Table S6.** The expression patterns of *M.officinalis* flavonol biosynthesis genes. **Table S7.** The level of flavonoid metabolites by Metabolome analysis. **Table S8.** Detailed data for Co-expression analysis of five MoMYB genes, flavonol biosynthesis genes, and flavonol metabolites in AR, TR, and SR samples. **Table S9.** Gene specific primers used in RT-qRCR.

## Data Availability

The sequenced raw reads generated during the current study have been submitted to the National Center for Biotechnology Information (NCBI) with BioProject ID: PRJNA717096 (https://www.ncbi.nlm.nih.gov/bioproject/?term=PRJNA717096).

## Abbreviations

MeJA: Methyl Jasmonate
ABA: Abscisic acid
PAL: Phenylalanine ammonialyase
CHI: Chalcone isomerase
4CL: 4-Coumarate-CoA ligase
CHS: Chalcone synthase
FLS: Flavonol synthase
F3H: Flavanone 3-hydroxylase
