# Supplementary material for: Genomic-wide identification and expression analysis of R2R3-MYB transcription factors related to flavonol biosynthesis in Morinda officinalis
Source: BMC Plant Biol. 2023 Aug 7;23:381. doi: 10.1186/s12870-023-04394-6 (PMC10405574; doi:10.1186/s12870-023-04394-6)
Supplement: Supplementary file 1 — Additional file 1: Figure S1. The phylogenetic tree, conserved motifs, and exon-intron structure of MoMYB proteins. Figure S2. Phylogenetic tree of R2R3-MYB proteins from M. officinalis, O. pumila, C. canephora and A. thaliana. Figure S3. The Cis-element analysis. Figure S4. RT-qPCR of the expression profile of MoMYB genes under hormonal treatments. [file 12870_2023_4394_MOESM1_ESM.docx]

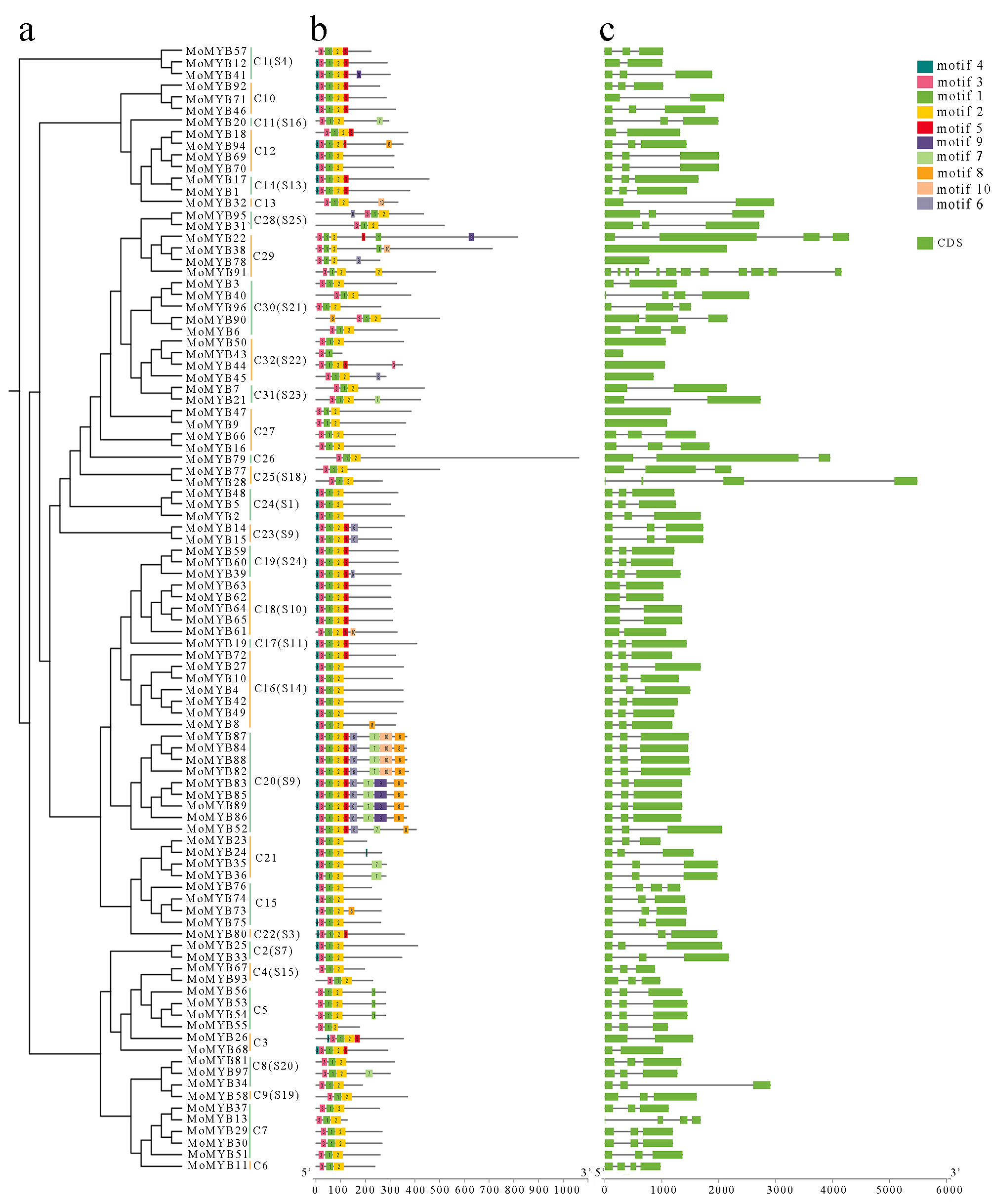


**Fig. S1.** The phylogenetic tree, conserved motifs, and exon-intron structure of MoMYB proteins. (A) The phylogenetic tree of MoMYB proteins. (B) The distribution of conserved motifs in MoMYB proteins. A total of 10 motifs were displayed using MEME with complete protein sequences. (C) The exon-intron structure of MoMYB genes. The grey line indicates intron; the green rectangle indicates exon.

**Fig. S2** Phylogenetic tree of R2R3-MYB proteins from *M. officinalis*, *O. pumila*, *C. canephora* and *A. thaliana*. The phylogenetic tree was created using IQ-tree with 1000 bootstrap replications. The model was VT+R8. The Phylogenetic tree is divided into 34 subfamilies, and the subfamilies are marked with tree branches of different colors, and numbers 1 to 34 are used to indicate the corresponding subfamily names in the outer circle. Known subfamily groups of Arabidopsis are distinguished by gene names with different background colors, marked as S1 to S25. The green five-pointed star, the blue circle, the red triangle and the orange square in the inner circle represent *M. officinalis*, *A. thaliana*, *O. pumila* and *C. canephora* protein, respectively.


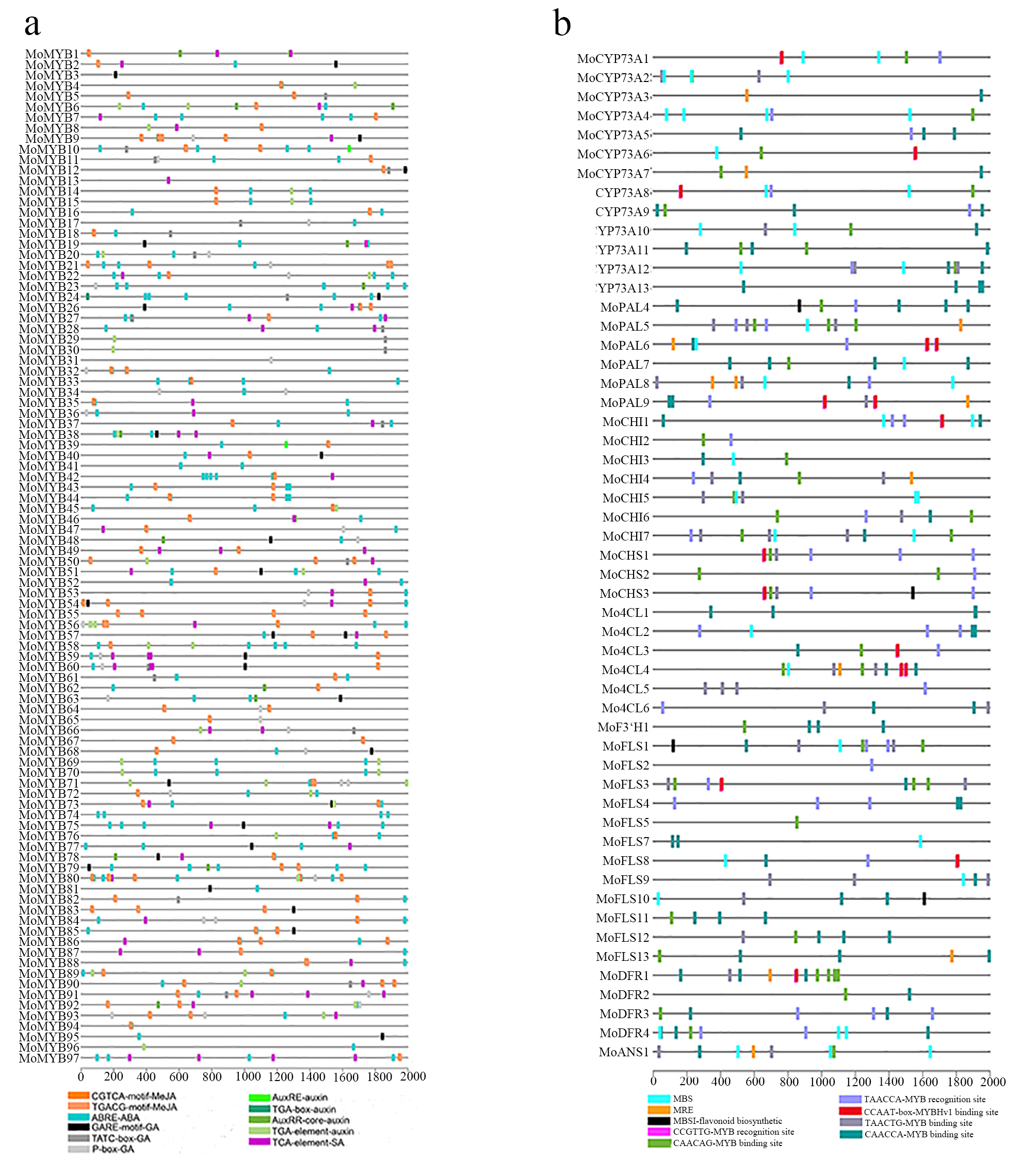


**Fig. S3** The *Cis-*element analysis. (**a**) Hormone-related *Cis-*element of the promoter of the MoMYB genes. (**b**) MYB-binding-related *Cis-*element of the promoter of the flavonoid biosynthesis genes.


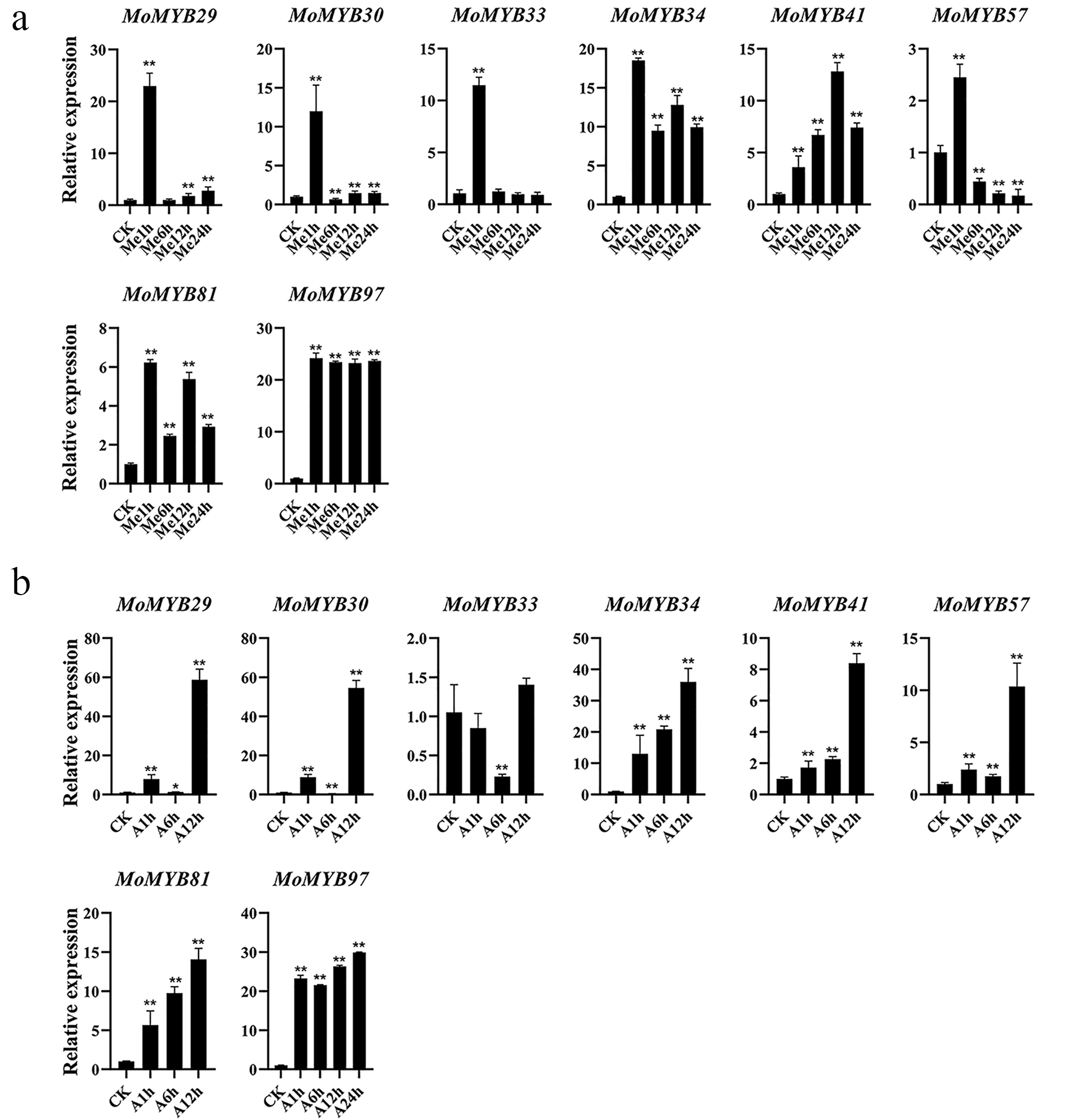


**Fig. S4** RT-qPCR of the expression profile of *MoMYB* genes under hormonal treatments. Me1h, Me6h, Me12h, and represent 1 h, 6 h, 12 h, and 24 h after MeJA treatment, respectively. A1h, A6h, and A12h represent 1 h, 6 h, and 12 h after ABA treatment. Statistically significant differences were determined by t-test (* p < 0.05, and ** p < 0.01).
